# Knowledge, Attitudes, and Practices of Pregnant Women and Hospital Staff Regarding Umbilical Cord Blood Banking: Systematic Review and Meta-Analysis

**DOI:** 10.3390/healthcare12212131

**Published:** 2024-10-25

**Authors:** Martina Benvenuti, Elisa Cavallini, Ginevra Battello, Fabrizio Zullo, Lorenza Driul, Antonella Cromi, Paolo Mannella, Rossella E. Nappi, Giovanni Scambia, Pasquale De Franciscis, Gaetano Riemma

**Affiliations:** 1 Division of Obstetrics and Gynaecology, Department of Clinical and Experimental Medicine, University of Pisa, 56124 Pisa, Italy; benvenuti.martina91@gmail.com (M.B.); paolo.mannella@unipi.it (P.M.); 2 Department of Obstetrics and Gynecology, University of Insubria, 21100 Varese, Italy; elisa.cavallini1993@gmail.com (E.C.); antonella.cromi@uninsubria.it (A.C.); 3 Medical Area Department (DAME), in Department of Medicine (DMED), University of Udine, 33100 Udine, Italy; ginevrabat@gmail.com (G.B.); lorenza.driul@uniud.it (L.D.); 4 Department of Maternal and Child Health and Urological Sciences, Sapienza University of Rome, 00185 Rome, Italy; f.zullo@hotmail.it; 5 Department of Clinical, Surgical, Diagnostic and Pediatric Sciences, University of Pavia, 27100 Pavia, Italy; rossella.nappi@unipv.it; 6 Research Center for Reproductive Medicine, Gynecological Endocrinology and Menopause, IRCCS San Matteo Foundation, 27100 Pavia, Italy; 7 Department of Women and Child Health, Division of Gynecologic Oncology, Fondazione Policlinico Universitario A. Gemelli IRCCS, 00168 Rome, Italy; giovanni.scambia@unicatt.it; 8 Department of Life Science and Public Health, Catholic University of Sacred Heart Largo Agostino Gemelli, 00168 Rome, Italy; 9 Department of Woman, Child and General and Specialized Surgery, University of Campania “Luigi Vanvitelli”, 80138 Naples, Italy; pasquale.defranciscis@unicampania.it

**Keywords:** umbilical cord blood, banking, storage, private umbilical cord blood storage, public umbilical cord donation

## Abstract

Background/Objectives: The aim of this study is to assess the knowledge, attitudes, and practices of pregnant women and hospital staff regarding umbilical cord blood (UCB) donation and storage to understand its limitations in clinical practice. Methods: MEDLINE, Scopus, LILACS, EMBASE, Scielo.br, and PROSPERO were searched from inception to 30 November 2023 with no geographic or language restrictions. The study eligibility criteria included cross-sectional studies that interviewed pregnant women and/or hospital staff about their knowledge, attitudes, and practices regarding private or public storage. A random-effects restricted maximum-likelihood model with Freeman–Tukey Double arcsine transformation meta-analysis was carried out to calculate the pooled estimates. MOOSE guidelines were followed. STATA 14.1 was used for statistical analysis. The Newcastle–Ottawa Scale and ROBINS-I tool were used for quality and risk of bias assessments. Results: In total, 19 studies providing data for 19,904 pregnant women and 1245 hospital staff members were included. Pooled pregnant women awareness was 61% ((95% CI 0.60 to 0.62), I^2^ = 0%, τ^2^ = 0.00, Q = 11.0 (*p* = 0.950)), and 61% for hospital staff (95% CI 0.58 to 0.64), I^2^ = 0%, τ^2^ = 0.00, Q = 4.00 (*p* = 0.310)). In total, 57% ((95% CI 0.56 to 0.58), I^2^ = 0, τ^2^ = 0.00, Q = 4.00 (*p* = 0.320)) of pregnant women had a positive attitude about UCB, while 34% ((95% CI 0.32 to 0.36), I^2^ = 0%, τ^2^ = 0.00, Q = 4.00 (*p* = 0.310)) were in favor of donating UCB for research and 65% ((95% CI 0.63 to 0.66), I^2^ = 0%, τ^2^ = 0.00, Q = 4.0 (*p* = 0.350)) were planning UCB storage. A significant (*p* < 0.001) preference for public relative to private banking (51% ([95% CI 0.49 to 0.54], I^2^ = 0%, τ^2^ = 0.00, Q = 4.0 (*p* = 0.310)) vs. 12% ([95% CI 0.10 to 0.13], I^2^ = 0%, τ^2^ = 0.00, Q = 4.0 (*p* = 0.300))) was noted for pregnant women. The same was retrievable for professionals (84% ([95% CI 0.79 to 0.88], I^2^ = 0%, τ^2^ = 0.00, Q = 2.0 (*p* = 0.110)) vs. 6% ([95% CI 0.03 to 0.09], I^2^ = 0%, τ^2^ = 0.00, Q = 1.0 (*p* = 0.070); *p* < 0.001)). Conclusions: Despite these efforts, lack of knowledge and positive attitudes about UCB banking remain, emphasizing the need for increasing educational programs on the subject.

## 1. Introduction

Umbilical cord blood (UCB) banking is an emerging practice in regenerative medicine, leveraging the rich source of hematopoietic stem cells found in UCB for the treatment of various hematological, genetic, and immune disorders [1]. UCB has been shown to be a viable alternative to bone marrow transplantation, offering similar or better outcomes in many cases, such as in leukemia and immunodeficiencies [2], and even emerging applications in treating high-incidence conditions such as diabetes [3], myocardial infarction [4], and neurodegenerative diseases [5].

Despite such advances, a significant gap in awareness and understanding among key stakeholders, including pregnant women and healthcare professionals, remains regarding the potential benefits and options for UCB banking [6]. Public banks, which are government-supported and rely on altruistic donation, play a critical role in ensuring a broader donor pool for hematopoietic stem cell transplants [7]. In contrast, private banks, which store UCB for potential future personal or familial use, are more controversial and are often debated in term of their necessity and cost effectiveness [8].

The need for enhanced education and training for both healthcare providers and potential donors is evident, as current levels of knowledge and positive attitudes towards UCB banking remain insufficient [9].

The American College of Obstetricians and Gynaecologists (ACOG), American Academy of Paediatrics (ACP), and American Society of Bone Marrow Transplant (ASBMT) also do not recommend private storage unless there is an identified need in the family in which banked cord blood would offer a benefit [10,11]. For better achievement of UCB collection, adequate education and training of obstetricians, midwives, and nurses to improve the awareness and the knowledge of parents is mandatory [12,13]. The recent literature shows a gap in the medical knowledge in this field and, consequently, a low awareness in pregnant women [14,15]. This lack of education is one of the most important causes of the loss of opportunity to collect and donate UCB [16].

This systematic review and meta-analysis aimed to assess the knowledge, attitudes, and practices of pregnant women and hospital staff regarding UCB donation and storage in order to identify key barriers and gaps in understanding, with the goal of informing strategies to improve awareness and participation in UCB banking. Moreover, we aimed to evaluate the attitudes around donating to public or private banks and the subsequent reasons for this choice.

## 2. Materials and Methods

The current meta-analysis was conducted in accordance with the Meta-analysis Of Observational Studies in Epidemiology (MOOSE) [17] standards.

Prior to article inspection, data extraction, tabulation, and analysis, the study protocol was created a priori, and we defined procedures for screening the literature, as well as the inclusion and exclusion criteria. On 30 November 2023, the study was registered in the database of the International Prospective Register of Systematic Reviews (CRD42023484499).

### 2.1. Eligibility Criteria

Studies were selected if they had a cross-sectional design and interviewed either pregnant women or other types of patients and/or healthcare workers about their awareness, knowledge, and attitude toward UCB or their knowledge or preference regarding private or public UCB storage.

Studies with unretrievable data or in which the questionnaires used were not publicly available were excluded from this analysis. Editorials, rebuttals, letters to the editor, and commentaries were also excluded.

### 2.2. Study Selection

After determining the inclusion criteria, two authors (GR and MB) examined every abstract and then every article in its entirety. Excluded from the analysis were papers that were redundant or had data that were replicated in later articles. A third author (P.D.F.) was consulted for further insight in the event of articles that raised questions.

When the approach suggested that more outcome data were reported, further unpublished data were collected by getting in touch with the original article’s authors.

### 2.3. Information Sources and Search Strategy

The following keywords and Medical Subject Heading (MeSH) terms were used to search eight electronic databases (MEDLINE (accessed through PubMed), Scopus, LILACS, EMBASE, Scielo.br, and PROSPERO) from the inception of each database to 30 November 2023: “umbilical cord blood” AND “banking” AND (“knowledge” OR “attitude” OR “information”).

Furthermore, we conducted searches in CINAHL, PsycINFO, and AMED to find additional pertinent publications and minimize publication bias.

To find additional studies not found using computerized searches, the reference lists of all qualifying publications and relevant reviews were also searched. There were no restrictions on language or region.

### 2.4. Assessment of Quality and Risk of Bias

Two different authors (GR and MB) assessed the risk of bias in all of the studies included using the ROBINS-I tool [18]. The ROBINS-I tool considers each study as an attempt to replicate a hypothetical pragmatic randomized trial and evaluates seven domains where bias could potentially be introduced: bias due to confounding, bias in the selection of participants into the study, bias in the classification of interventions, bias due to deviations from intended interventions, bias due to missing data, bias in the measurement of outcomes, and bias in the selection of the reported results. The judgments within each domain contribute to an overall assessment of bias, categorized as follows: “Low risk of bias”, “Moderate risk of bias”, “Serious risk of bias”, “Critical risk of bias”, or “No information” [18].

The modified Newcastle–Ottawa Scale (NOS) was utilized to evaluate the quality of observational research [19]. Every study was evaluated based on three fundamental components, as per the Newcastle–Ottawa Scale (NOS): the identification of the desired goal, the selection and comparability of study groups, and this process [19]. Evaluation of the research selection was composed of the following: determining the exposed cohort’s representativeness, choosing the non-exposed cohort, confirming the exposure, and confirming that the outcome under investigation was unlikely to arise on its own at the start of the study.

Study comparability was evaluated, and cohort comparability based on design or analysis was also evaluated. Additionally, the procedure for figuring out the outcome of interest, length, and sufficiency of follow-up were used to evaluate the ascertainment of the outcome of interest. For every numbered item in the selection and outcome categories, research received up to one star based on the NOS criteria.

### 2.5. Data Extraction

The data extraction form was specifically designed for this meta-analysis. The key characteristics recorded included pregnant women and hospital staff descriptors (parity, gestational age, marital status, education, and income for pregnant women, and qualification, specialization, years of practice, and gender for hospital staff), study duration, setting, type of cross-sectional assessment, features of the cohort and questionnaires, outcomes evaluated, quality elements, and main conclusions.

### 2.6. Statistical Analysis

A meta-analysis of proportions was performed using STATA vers. 14.1 (StataCorp LLC, College Station, TX, USA), with an alpha level of 0.05 set for statistical significance. Due to anticipated heterogeneity, the metaprop function was employed to combine proportions—defined as the number of events over the total number of observations—using a random-effects-restricted maximum-likelihood model (REML). When multiple proportions within a single study met the criteria for the primary outcome, only the highest proportion was included to avoid data duplication or distortion of the true effect size. If studies presented data for multiple follow-up questionnaires, the earliest were chosen for the primary analysis.

To stabilize variances, individual proportions were converted using the Freeman–Tukey Double arcsine transformation (FTT). Confidence intervals (CIs) for individual studies were computed using Clopper–Pearson 95%, while Wald 95% CIs were used for pooled proportions. Forest plots for each outcome were generated via the metaprop function using the command <metaprop event n, ftt>. Pre-specified random-effects subgroup analyses were conducted for variables, including the region of the study and education level and income of the sampled cohorts, with inter-group effect size differences evaluated using the Wald-type χ^2^ test. For all of the analyses, a *p*-value (*p*) less than 0.05 was considered statistically significant.

Heterogeneity was measured using the Higgins I^2^ statistic [20], with thresholds of 25%, 50%, and 75% corresponding to moderate, substantial, and considerable heterogeneity, respectively, and using Cochran Q’ test with its related *p*-value and τ^2^ statistics, as outlined in the *Cochrane Handbook for Systematic Reviews of Interventions* [20]. To detect publication bias, when the number of studies reporting the outcome of interest was at least 10, the Egger regression intercept test [21] and the Begg rank correlation test were applied [22].

### 2.7. Study Outcomes

The primary outcome of this meta-analysis was the UCB patient awareness rate, defined as the percentage of interviewed participants on overall participants that were aware of the possibility of UCB banking before the interview [23].

Secondary outcomes included the following: positive attitude rate, defined as the frequency of interviewed participants that had a positive opinion of UCB donation; good knowledge rate, defined as frequency of interviewed participants that had a good knowledge of UCB possibility before the interview; patient preference, defined as the reported preference of women to store UCB in a public or private bank; healthcare workers preference, defined as the reported preference of healthcare professionals to store UCB in a public or private UCB bank; research donation, defined as the percentage of interviewed women who would donate due to research purposes; and plan-to-donate rate, defined as the percentage of interviewed participants who planned to donate their UCB after delivery.

## 3. Results

### 3.1. Study Selection

**Figure 1:**
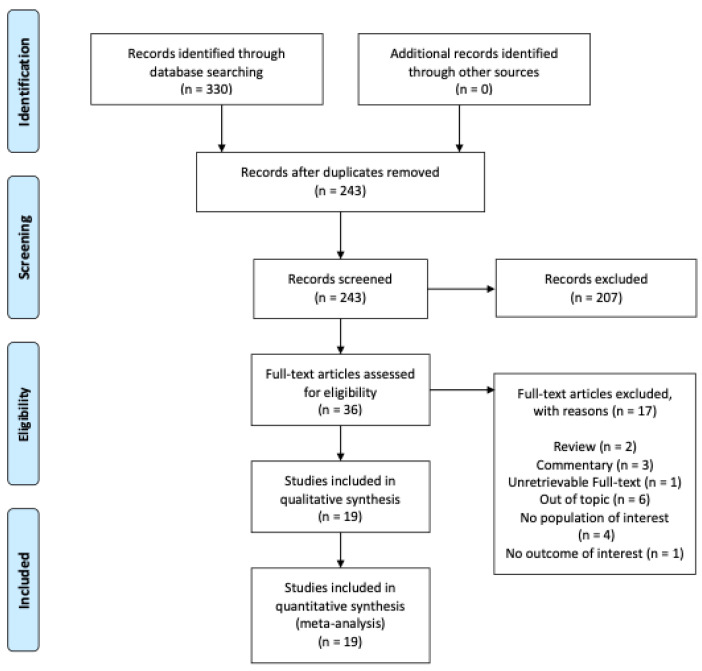
Flowchart of studies identified for systematic review and meta-analysis.

Initially, 330 studies were identified through a database search. Of those, 87 were removed as duplicates. After title and abstract screening, 36 papers were identified and underwent a full-text assessment. Subsequently, six papers were removed for being out-of-topic, three for being commentaries, two for being reviews, four for not reporting populations of interest, and one for not reporting outcomes of interest. In total, 19 studies [23,24,25,26,27,28,29,30,31,32,33,34,35,36,37,38,39,40,41] with 19,904 pregnant women and 1245 hospital staff members were included in this quantitative synthesis and meta-analysis. Figure 1 and Supplementary File S1 depict the flow of the study selection process.

### 3.2. Study Characteristics

The main characteristics of the included studies and related survey information are reported in Table 1.

Most studies were conducted in Europe (7/19; 37%), followed by North America (6/19; 32%) and Asia (4/19; 21%), with one study each carried out in Oceania (1/19; 5%) and South America (1/19; 5%), respectively (Table 1).

Fourteen studies surveyed pregnant women, while seven analyzed opinions from hospital staff (obstetricians, pediatricians, nurses, technicians, and midwifes).

Five studies ascertained differences between private and public UCB banking among pregnant women, while three studies surveyed healthcare providers regarding the same topic (Table 2). One study evaluated differences in UCB knowledge between urban and rural areas. A detailed questionnaire assessment is reported in Supplementary File S2.

### 3.3. Risk of Bias of Included Studies

The risk of bias, measured by means of the ROBINS-I tool, showed that most of the studies had an overall low risk of bias, with only 4 out of 18 studies categorized as moderate risk [26,35,37,41]. A detailed assessment is reported in Table S1.

Based on the Newcastle–Ottawa Scale criteria, all studies found high scores, with a minimum of seven and a maximum of nine, for quality. Based on controls for age, education level, and occupation as the three jointly most relevant criteria, the comparability of cohorts, whereas needed, achieved the maximum score. Table S2 provides a thorough point-by-point evaluation.

Publication bias for the primary outcome, awareness of UCB banking in pregnant women, as measured by Egger’s and Begg’s tests (Egger’s test *p* = 0.754 and Begg’s test *p* = 0.891) and funnel plot analyses (Figure S1a), was not apparent. The assessment of publication bias for all the secondary outcomes was not feasible, since the number of studies evaluating each outcome was fewer than ten.

### 3.4. Synthesis of Results

#### 3.4.1. Primary Outcome: Awareness of UCB by Pregnant Women

**Figure 2:**
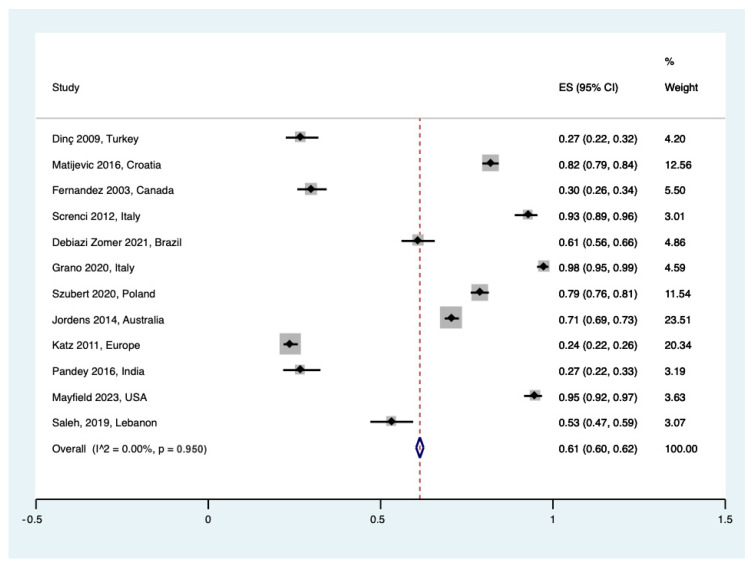
Forest plot for awareness of UCB banking for patients [23,25,27,30,31,33,34,35,38,39,40,41].

Overall, 14 studies evaluated the awareness rate of banking umbilical cord blood at delivery among pregnant women. Pooled awareness was 61% ((95% CI 0.60 to 0.62), I^2^ = 0%, τ^2^ = 0.00, Q = 11 (*p* = 0.950)). No significant heterogeneity was reported (Figure 2).

Divided by study region, the results show that pregnant women’s awareness rate reached its maximum in North America (73% (95% CI 0.71 to 0.75), I^2^ = 0%, τ^2^ = 0.00, Q = 2.00 (*p* = 0.100)), while the pooled awareness rate reached its minimum in Asia (39% (95% CI 0.35 to 0.44, I^2^ = 0%, τ^2^ = 0.00, Q = 1.00 (*p* = 0.070)) (Figure S2).

#### 3.4.2. Awareness of UCB Banking by Hospital Staff

**Figure 3:**
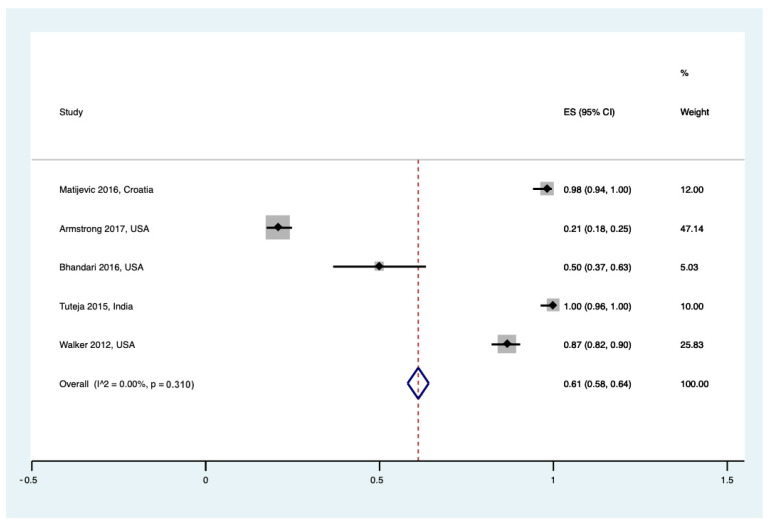
Forest plot for awareness of UCB banking for hospital staff [24,25,26,28,29].

Regarding hospital staff, data about awareness of UCB banking were reported in five studies. The pooled awareness rate was similar to the one reported by pregnant women (61% (95% CI 0.58 to 0.64), I^2^ = 0%, τ^2^ = 0.00, Q = 4.00 (*p* = 0.310)) (Figure 3).

Subgroup analysis by region was not achievable since three out of five studies were carried out in the US, while the other two were from Croatia and India, respectively.

#### 3.4.3. Positive Attitude Toward UCB Banking

**Figure 4:**
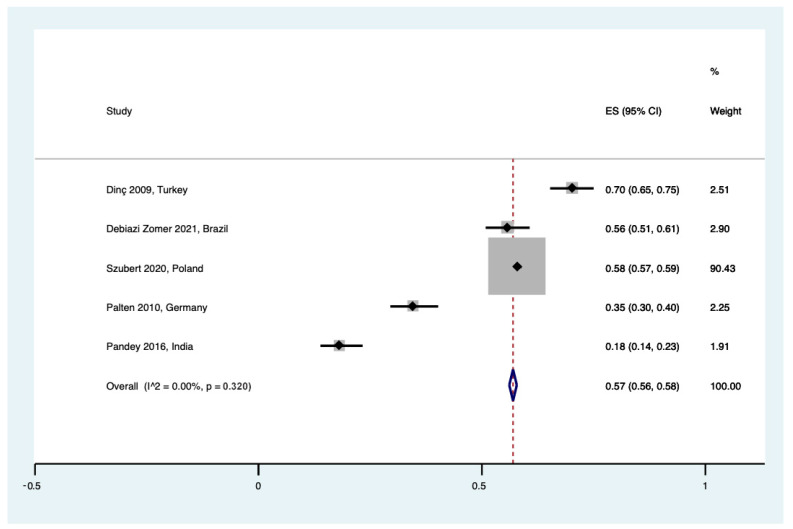
Forest plot for positive attitude regarding UCB banking [23,27,37,38,41].

Six studies surveyed pregnant women regarding their opinion on UCB banking before receiving counseling. The evaluated pooled result for a positive attitude on banking UCB was 57% ((95% CI 0.56 to 0.58), I^2^ = 0, τ^2^ = 0.00, Q = 4.00 (*p* = 0.320)) (Figure 4), showing that there is a minor percentage of uncertainty and less trust regarding UCB banking.

There were no studies reporting a positive or negative attitude on UCB banking for hospital staff.

#### 3.4.4. Research Purposes

**Figure 5:**
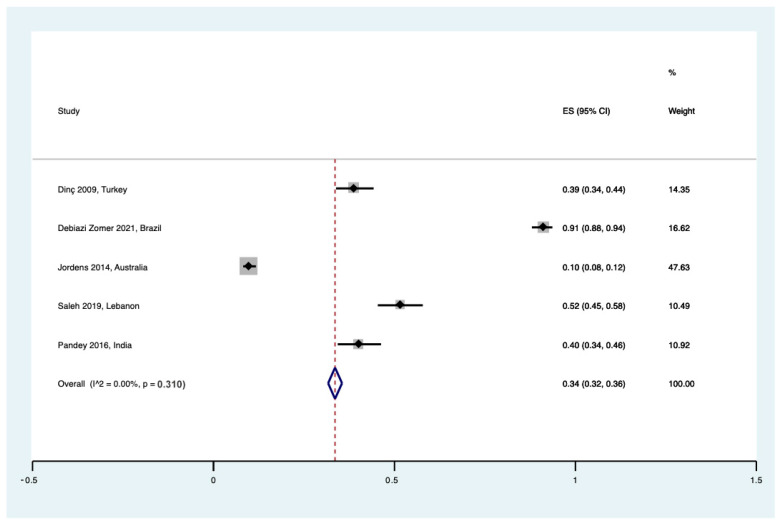
Forest plot for research purposes donation regarding UCB for pregnant women [23,27,33,35,38].

Pregnant women were questioned about the possibility of donating UCB for research or scientific purposes in seven studies. Interestingly, less the 50% of women reported in favor, with a pooled prevalence of 34% ((95% CI 0.32 to 0.36), I^2^ = 0%, τ^2^ = 0.00, Q = 4.00 (*p* = 0.310)) (Figure 5).

There was no data available data for the hospital staff cohort.

#### 3.4.5. Planning to Store UCB

**Figure 6:**
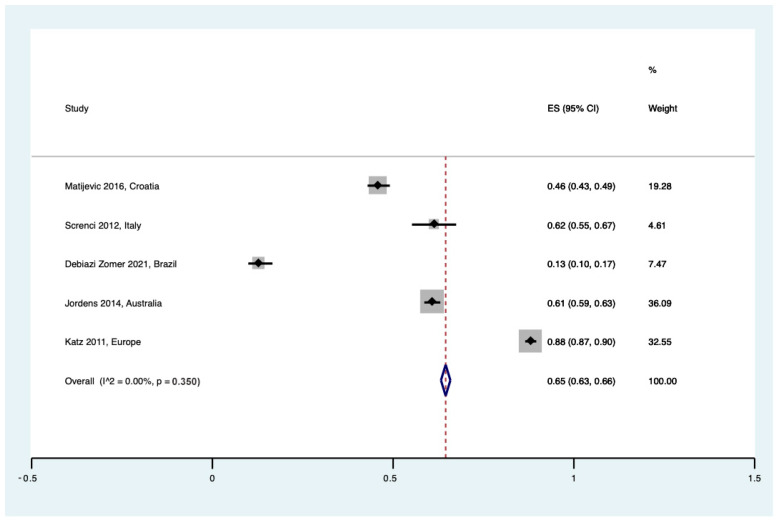
Forest plot showing those in favor of donation for planning to store UCB [25,27,31,33,34].

Answers regarding whether patients were planning to store UCB in their upcoming pregnancy were noted in five studies. Overall, there was a moderate willingness to store UCB, with a pooled incidence of 65% ([95% CI 0.63 to 0.66], I^2^ = 0%, τ^2^ = 0.00, Q = 4.0 (*p* = 0.350)) (Figure 6).

#### 3.4.6. Public vs. Private UCB Storage

Pregnant women and hospital staff members were also asked about their preference in choosing or advising a public or private bank for UCB storage.

**Figure 7:**
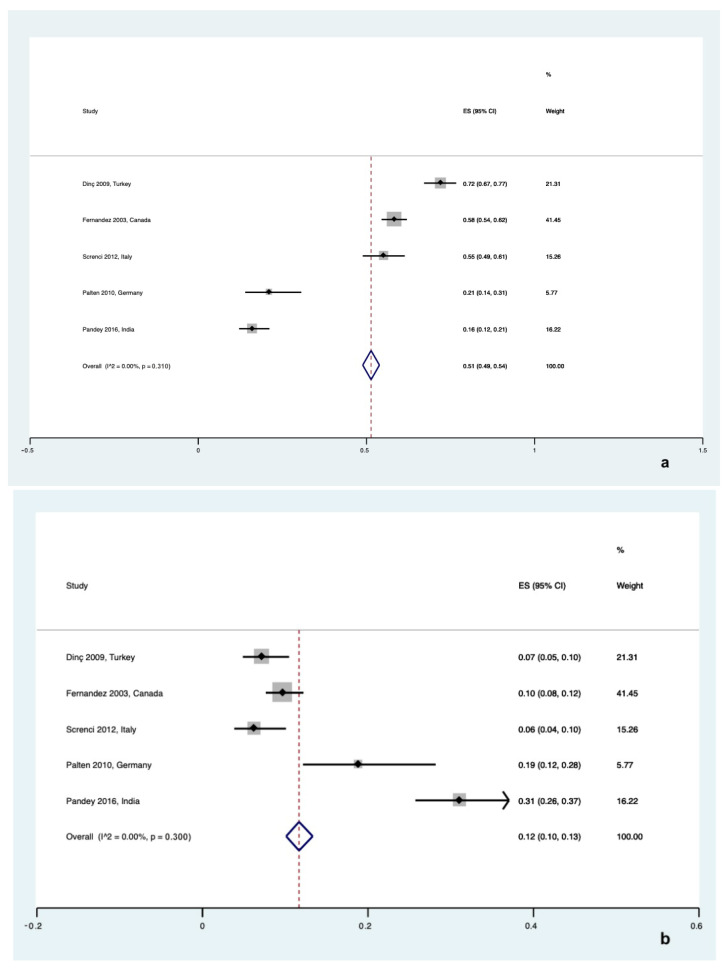
Forest plot for preference in choosing a public or private bank for UCB storage in (**a**) pregnant women [23,30,31,37,38] and (**b**) hospital staff [23,30,31,37,38].

Five studies surveyed the preference of pregnant women, showing a significant preference for public UCB banking with a pooled rate of 51% ([95% CI 0.49 to 0.54], I^2^ = 0%, τ^2^ = 0.00, Q = 4.0 (*p* = 0.310)) compared to a pooled rate of 12% ([95% CI 0.10 to 0.13], I^2^ = 0%, τ^2^ = 0.00, Q = 4.0 (*p* = 0.300)) (*p* < 0.001). (Figure 7a,b).

**Figure 8:**
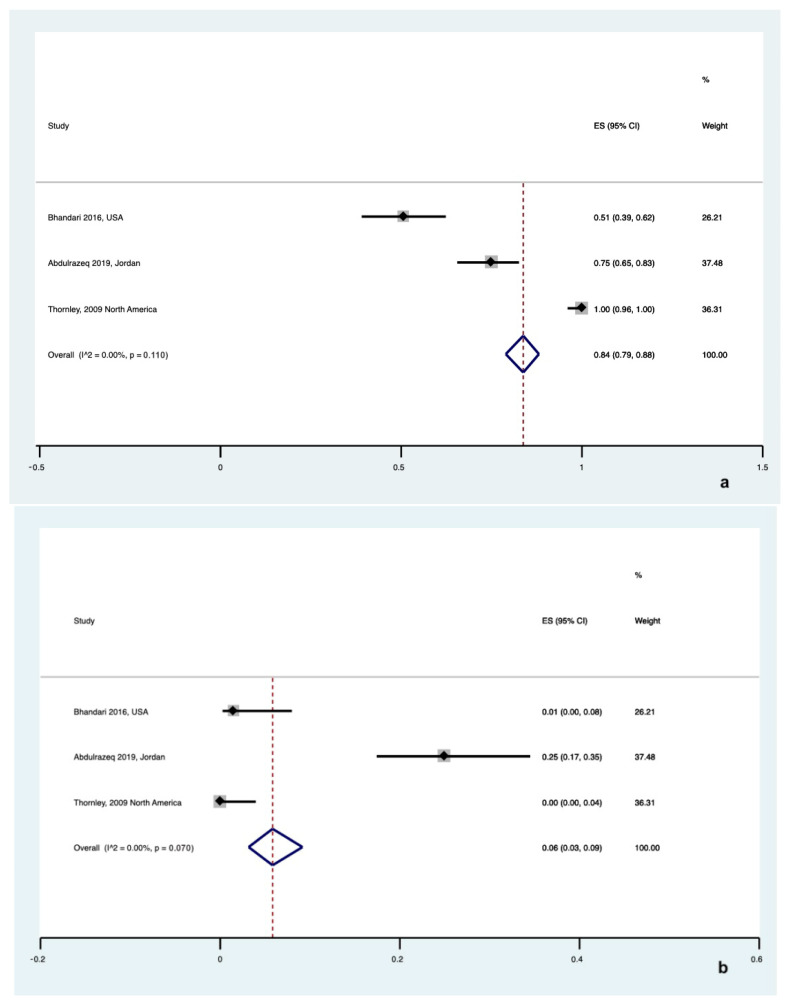
Forest plot for preference in choosing a (**a**) public [26,32,36] or (**b**) private bank for UCB storage in hospital staff [26,32,36].

The opinions of hospital staff on choosing a private vs. a public UCB bank were collected in three studies only. Most of the hospital staff were favorable to public banking (pooled rate 84% [95% CI 0.79 to 0.88], I^2^ = 0%, τ^2^ = 0.00, Q = 2.0 (*p* = 0.110)), while only 6% ([95% CI 0.03 to 0.09], I^2^ = 0%, τ^2^ = 0.00, Q = 1.0 (*p* = 0.070)) preferred to choose a private UCB bank (*p* < 0.001) (Figure 8a,b).

#### 3.4.7. Subgroup Analysis

Subgroup analysis of specific categories was performed to seek out differences among substrates of pregnant women and hospital staff. The subgroup analysis for pregnant women is reported in Table 3.

Pregnant women younger than 30 years old showed a more positive attitude and more interest in donating their UCB for research compared to older women (Table 3; *p* < 0.001). Conversely, more intention to donate UCB was observed in older women relative to women under 30 years (Table 3; *p* < 0.001).

Interestingly, studies conducted after 2015 reported increased awareness and research purpose rates compared to studies conducted before 2015 (Table 3; *p* < 0.001). Conversely, pregnant women willing to donate UCB were diminished (Table 3; *p* < 0.001).

Concerning the hospital staff, for the awareness rate, the mean age was reported in one study only, while the education level (university or college) and area of residence (urban) were the same in all of the included studies, showing no differences relative to the overall analysis.

Regarding the choice of a public UCB banking, the subgroup analysis in pregnant women showed similar estimates for age under or over 30 (53% [95% CI 0.50 to 0.56], I^2^ = 0%, τ^2^ = 0.00, Q = 2.0 (*p* = 0.110) vs. 55% [95% CI 0.49 to 0.61], I^2^, τ^2^, Q = NE)), and education level (high school or less vs. university or college) (49% [95% CI 0.46 to 0.53], I^2^ = 0%, τ^2^ = 0.00, Q = 2.0 (*p* = 0.100) vs. 54% [95% CI 0.54 to 0.57], I^2^ = 0%, τ^2^ = 0.00, Q = 1.0 (*p* = 0.070)).

Considering the minority of pregnant women who preferred private UCB banking, no differences were found regarding age (12% [95% CI 0.11 to 0.14], I^2^ = 0%, τ^2^ = 0.00, Q = 2.0 (*p* = 0.110) vs. 6% [95% CI 0.04 to 0.10], I^2^, τ^2^, Q = NE)), and education level (13% [95% CI 0.11 to 0.15], I^2^ = 0%, τ^2^ = 0.00, Q = 2.0 (*p* = 0.100) vs. 11% [95% CI 0.08 to 0.13], I^2^ = 0%, τ^2^ = 0.00, Q = 1.0 (*p* = 0.070)). For both private and public banking preferences, a subgroup analysis for area of residence was not carried out due to the lack of studies, with most pregnant women coming from rural areas.

Due to the paucity of studies reporting data for a public or private preference for hospital staff and their related characteristics, it was not feasible to carry out the subgroup analysis for this outcome.

## 4. Discussion

The results of this systematic review and meta-analysis provide a comprehensive overview of UCB banking among pregnant women and hospital staff. The analysis showed a pooled overall awareness rate of about 60% for both pregnant women and hospital staff members, with higher awareness in Europe and North America and less in Asian countries. Considering the higher rates in studies conducted after 2015, a positive trend in reducing the knowledge gap about UCB, its banking, and usefulness is foreseeable. A generally positive attitude in regions with higher awareness levels, such as North America and Europe, was also reported. However, studies from countries with lower awareness, e.g., several Asian countries, reported more neutral or negative attitudes. This correlation between awareness and attitude suggests that targeted educational programs could significantly influence both pregnant women and hospital staff behavior towards UCB banking.

The access to information about the topic is, indeed, significantly greater in Europe and North America. However, we performed a subgroup analysis on the educational status of the population involved in the several surveys that highlighted a different stratification of awareness and knowledge. According to this analysis, a survey conducted in Missouri (USA) showed that respondents with education beyond high school were more aware of the concept of UCB collection compared with their less-educated counterparts. In additional, the last group is prevalently composed of ethnic minorities [39]. An Italian survey conducted in 2020 also emphasized the positive relationship between schooling, age, and awareness about UCB banking [40]. A recent Polish study compared the awareness and knowledge of pregnant women in urban and rural areas, showing that levels of knowledge about UCB banking among pregnant women living in urban areas were higher than among those in rural areas [41].

Surprisingly, more willingness to donate UCB was found in pregnant women without a degree. This could be related to the increased levels of skepticism of such patients, as well as a plausible non-scientific degree (e.g., humanities) of these women, which are data we were unable to collect in the original studies [42].

The awareness of hospital staff members appears similar to what reported by pregnant women. Studies focusing on hospital staff reported a basic know-how regarding the usefulness of UCB banking, but most of hospital staff members considered their knowledge insufficient. This could be related to a lack of information and specific education in healthcare providers. In an American survey, only 11% of physicians received formal education about UCB banking [28]. Moreover, the confidence in this field changes according to healthcare role (obstetrician, pediatric, midwife, nurse), years of practice, and hospital affiliation to UCB banks [25,28,29].

The meta-analysis highlights a low prevalence of people in favor of research purpose. The poor availability to donate UCB for research purposes seems to be related to a lack knowledge and/or to a preference for private UCB banking, as shown by an Australian survey [18]. Moreover, in some countries, the opposition to storing UCB for studying purposes had ethical, religious, or ideological origins [23,35].

This meta-analysis also highlights the preference for public over private UCB banking in most regions, which aligns with recommendations from leading health organizations [9,10,11]. However, this preference is not universal; some populations, particularly in areas where private banking is heavily marketed, still show a notable preference for private options. These findings suggest that while public UCB banking is broadly favored, cultural, economic, and marketing influences continue to shape individual preferences [6,8].

Public banking is widely encouraged by medical professionals due to its broader social benefits and ethical advantages [9]. Unlike embryonic stem cells, UCB stem cells do not raise significant ethical concerns, and their collection does not pose a risk to the mother or the child. Nonetheless, the efficacy of UCB in treating a wide range of conditions remains a topic of ongoing research. Therefore, experts caution against banking for unproven future uses and emphasize the importance of realistic expectations. People who decide to collect their child’s UCB in private banks usually fear the onset of illness in their own children or family. As a matter of fact, this attitude can be observed in countries where there is a high percentage of interfamily marriages, such as Turkey [23]. However, to date, the real advantage of this choice is questionable: the real risk in needing to own stem cells over their lifetime is very low; moreover, some hematologic illnesses benefit more from allogenic rather than autologous transplantation [30,38]. In this scenario, more emphasis should be placed for promoting an increase in UCB donations [1].

The surveys of the hospital staff members regarding UCB banks show a prominent preference for public banking, as hospital staff working in public hospitals tend to have greater confidence in different storage modalities and purposes [29]. However, there are no data available regarding their preferences in terms of research and social purposes, reflecting a need to perform additional studies and educational program assessments that could help improve awareness on the topic and, consequently, enhance patient counseling [26,36]. Providing more awareness does not seem to discourage the decision to bank umbilical cord blood; instead, it tends to make those who are undecided more likely to choose this option. Additionally, it can have a similar effect on about half of those who have not yet considered it [33]. Parental choices regarding umbilical cord blood banking can be influenced by “media hype”, marketing by commercial entities, fears about illness, hopes for future medical breakthroughs, and a desire to protect their children. The information given to prospective parents about umbilical cord blood banking should be accurate, balanced, and comprehensive, outlining both the benefits and drawbacks of donation and storage, so parents can make well-informed decisions [6]. This includes clarifying that the chances of the stored sample being used to treat the donor’s own child are currently minimal, that future therapeutic possibilities remain highly speculative, and that there is no current evidence suggesting that ongoing research will lead to specific therapeutic applications for an individual’s own cord blood cells [2,5,6,13]. Such issues are corroborated by the reduced incidence of women willing to donate UCB in more recent studies (conducted after 2015). Therefore, the information provided should be accurate and specifically address common misconceptions.

Several limitations should also be acknowledged about this systematic review and meta-analysis. Its main limitation is related to the intrinsic baseline differences among the analyzed population, even without significant heterogeneity. To lessen this effect, the meta-analysis was conducted using a random-effect model and several subgroup analyses. It is recommended that the substantial variability may be attributed to changes in sample size, methodological evaluation, demographic and intrinsic characteristics of the research populations, and study durations that differ between studies. Moreover, even if overall good scores in quality and risk of bias assessments were achieved, the cross-sectional design of all of the included studies reduces the certainty of evidence to low. Additionally, the variability between the questionnaires among the studies inevitably limits the reliability of the estimates. To mitigate this effect and ensure consistency, we provided the original questions used for each outcome’s evaluation as Supplementary Material (Supplementary File S2). Lastly, although the publication bias, as measured using Egger’s and Begg’s tests and funnel plot asymmetry analyses, was not apparent for the primary outcome, we were unable to assess it for the other evaluated outcomes due to the limited (less than ten) number of studies reporting analyzable data. Indeed, with fewer studies, the power of the tests might be too low for distinguishing real asymmetries. Therefore, for such outcomes, although no substantial heterogeneities were found, the possibility of a small-study effect should not be excluded [42].

However, several points of strength should be highlighted. Firstly, no significant heterogeneity was reported for the primary outcome and all of the other secondary outcomes of this review. This should be related to the coherence of the study populations, which was limited to two different cohorts that were analyzed separately (pregnant women and hospital staff). All of the studies including non-pregnant women or other subgroups of patients were excluded from this analysis. In fact, although it could be argued that it is difficult to believe that heterogeneity is 0 for all of the analyses of the different forest plots, when the study population is not heterogenous and the random-effect model with Freeman–Tukey arcsine transformations are used to stabilize the variance, it is common to retrieve low heterogeneity [43]. Such an approach is highly recommended and remains the preferred methodology for the analysis of data involved in meta-analyses of proportions [44].

Secondly, to date, this is the first systematic review and meta-analysis that summarizes the available literature on pregnant patients’ and hospital staff’s perspectives on UCB banking, reducing a gap in the current literature, which represents an additional strength of this study.

## 5. Conclusions

Despite efforts taken to increase the awareness of UCB collection, a clear lack of knowledge and positive attitudes remains in both pregnant women and hospital staff. Health professionals and organizations should step up their efforts to spread knowledge and support educational programs regarding the donation and storage of UCB for research and social purposes. Giving pregnant parents more detailed information about UCB may boost their satisfaction with the information they know about it and may change their opinions regarding cord blood donation with the aim of helping to cure several immunologic, hematologic, and neurodegenerative pathologies.

## Figures and Tables

**Table 1:** Characteristics and methodologies of available UCB knowledge surveys.

Study	Year	Location	Population (Pregnant Women or Hospital Staff)	Methodology	Age, Mean or Median (SD or IQR)	Education (>60% of Sample)	Area of Residence, (>60% of Sample)	Awareness Rate (%)	Positive Attitude Rate (%)	Research Purpose Rate (%)	Plans to Store UCB Rate (%)
Dinc [23]	2009	Turkey	334 pregnant women	Interview form developed by the researchers according to the literature	26.5 (4.9)	High school diploma or lower	Urban area	90/334 (26.9)	235/334 (70.3)	130/334 (38.9)	NA
Matijevic [25]	2016	Croatia	1000 pregnant women and 120 workers in hospital maternity staff (nurses, midwives, trainees and specialists in gynecology and obstetrics)	Two anonymous questionnaires	NA	NA	Urban area	Pregnant women 820/100 (82.0) Hospital staff 118/120 (98.3)	NA	NA	460/1000 (46.0)
Fernandez [30]	2003	Canada	650 pregnant women	Anonymous questionnaires	29 (12–44)	University or college	NA	131/438 (29.9)	NA	NA	NA
Armstrong [28]	2017	USA	473 pediatric medical providers (medical students, residents, advanced nurse practitioners, subspecialists, hospitalists, academic general pediatricians, and private practice pediatricians)	Questionnaire composed of 26 questions	35 (10)	University or college	Urban area	99/473 (21.0)	NA	NA	NA
Bhandari [26]	2016	USA	67 providers as nurse/nurse practitioners (*n* = 17) and physicians (*n* = 50); 222 pregnant women	Survey questionnaires	NA	University or college	Urban area	25/50 (50.0)	NA	NA	NA
Screnci [31]	2012	Italy	300 pregnant women	Two types of anonymous questionnaires	40 (18–63)	High school diploma or lower	NA	222/239 (92.8)	NA	NA	147/239 (61.5)
Debiazi Zomer [27]	2021	Brazil	387 pregnant women	Questionnaire-based study	30 (NA)	High school diploma or lower	NA	236/387 (60.9)	216/387 (55.9)	353/387 (91.2)	50/387 (12.9)
Grano [40]	2020	Italy	365 pregnant women in the third trimester of pregnancy	Questionnaire	33.2 (4.2)	University or college	NA	356/365 (97.5)	NA	NA	NA
Szubert [41]	2020	Poland	12066 pregnant women	Double questionnaire completed by the pregnant women	30 (NA)	University or college	Urban area	725/919 (78.9)	NA	NA	NA
Abdulrazeq [32]	2019	Jordan	96 obstetricians	Questionnaire	35.7 (11.1)	University or college	Urban area	NA	NA	NA	NA
Jordens [33]	2014	Australia	1873 pregnant women	Survey questionnaires	27 (7)	University or college	Urban area	1324/1873 (70.7)	NA	109/1110 (9.8)	NA
Katz [34]	2011	Europe (France, Germany Italy, Spain, UK)	1620 pregnant women	Anonymous and self-completed questionnaire	32 (NA)	High school diploma or lower	Urban area	386/1620 (20.6)	NA	NA	NA
Saleh [35]	2019	Lebanon	244 pregnant women	Descriptive study with questionnaire	28.8 (6.6)	University or college	Urban area	130/244 (53.9)	NA	126/144 (51.6)	NA
Thornley [36]	2009	USA and Canada	130 eligible pediatric physicians	Emailed cross-sectional survey	NA	University or college	Urban area	NA	NA	NA	NA
Palten [37]	2010	Germany	300 pregnant women	Questionnaires	NA	University or college	Urban area	NA	7000/12066 (58.0)	NA	NA
Pandey [38]	2016	India	254 pregnant women	Explorative questionnaire-based survey	25.0 (5.0)	High school diploma or lower	NA	68/254 (26.5)	46/254 (18.1)	102/254 (40.1)	NA
Mayfield [39]	2023	USA	289 pregnant women	Survey using research electronic data capture	21.0 (13.5)	High school diploma or lower	NA	274/289 (94.8)	NA	NA	NA
Tuteja [24]	2015	India	100 physicians	Objective questionnaire	NA	University or college	NA	100/100 (100)	NA	NA	NA
Walker [29]	2012	USA	259 obstetricians	Mailed survey	NA	University or college	Urban area	225/259 (86.9)	NA	NA	NA

NA: not available.

**Table 2:** Public vs. private UCB banking in included studies.

Study	Year	Population	In Favor of Public Banking (%)	In Favor of Private Banking (%)
Dinc [23]	2009	Pregnant women	241/334 (72.1)	24/334 (7.1)
Fernandez [30]	2003	Pregnant women	379/650 (58.3)	63/650 (9.6)
Bhandari [26]	2016	Hospital staff	34/67 (50.7)	1/67 (1.5)
Screnci [31]	2012	Pregnant women	132/239 (55.2)	15/239 (6.2)
Abdulrazeq [32]	2019	Hospital staff	72/96 (75.0)	24/96 (25.0)
Thornley [36]	2009	Hospital staff	93/93 (100)	0/93 (0)
Palten [37]	2010	Pregnant women	19/90 (21.1)	17/90 (18.8)
Pandey [38]	2016	Pregnant women	41/254 (16.1)	79/254 (31.1)

**Table 3:** Subgroup analysis of study outcomes for pregnant women.

	Awareness Rate		Positive Attitude Rate Positive Attitude Rate		Research Purpose Rate		Plans to Store UCB Rate Plans to Store UCB Rate	
Subgroup	Studies	Pooled rate, % (95% CI, I^2^, τ^2^, Q (*p*))	No. of studies	ww Pooled rate, % (95% CI, I^2^, τ^2^, Q (*p*))	Studies	Pooled rate, % (95% CI, I^2^, τ^2^, Q (*p*))	Studies	Plans to store UCB rate (%) Pooled rate, % (95% CI, I^2^, τ^2^, Q (*p*))
Mean age Equal or over 30 under 30	5 6	60 (0.58 to 0.61, I^2^ = 0%, τ^2^ = 0.00, Q = 4.0 (*p* = 0.320)) 61 (0.60 to 0.62; I^2^ = 0%, τ^2^ = 0.00, Q = 5.0 (*p* = 0.400))	2 2	47 (0.43 to 0.51, I^2^ = 11%, τ^2^ = 0.00, Q = 1.0 (*p* = 0.070)) 58 (0.57 to 0.59, I^2^ = 0%, τ^2^ = 0.00, Q = 1.0 (*p* = 0.100))	3 2	29 (0.26 to 0.31, I^2^ = 0%, τ^2^ = 0.00, Q = 2.0 (*p* = 0.100)) 43 (0.40 to 0.46, I^2^ = 0%, τ^2^ = 0.00, Q = 1.0 (*p* = 0.310))	3 1	75 (0.73 to 0.77, I^2^ = 0%, τ^2^ = 0.00, Q = 2.0 (*p* = 0.100)) 61 (0.59 to 0.63; I^2^, τ^2^, Q, *p* = NE)
Education level High school or less University or College	6 5	42 (0.40 to 0.43, I^2^ = 0%, τ^2^ = 0.00, Q = 5.0 (*p* = 0.409)) 61 (0.60 to 0.62; I^2^ = 0%, τ^2^ = 0.00, Q = 4.0 (*p* = 0.310))	3 2	50 (0.47 to 0.54, I^2^ = 0%, τ^2^ = 0.00, Q = 2.0 (*p* = 0.190)) 57 (0.57 to 0.58, I^2^ = 0%, τ^2^ = 0.00, Q = 1.0 (*p* = 0.080))	2 2	63 (0.60 to 0.66, I^2^ = 0% τ^2^ = 0.00, Q = 1.0 (*p* = 0.060)) 16 (0.14 to 0.18, I^2^ = 0%, τ^2^ = 0.00, Q = 1.0 (*p* = 0.060))	3 1	75 (0.73 to 0.77, I^2^ = 0%, τ^2^ = 0.00, Q = 2.0 (*p* = 0.110)) 61 (0.59 to 0.63, I^2^, τ^2^, Q, *p* = NE)
Area of residence Urban Rural	6 0	59 (0.57 to 0.60, I^2^ = 0%, τ^2^ = 0.00, Q = 5.0 (*p* = 0.400)) /	3 0 3	58 (0.57 to 0.59, I^2^ = 0%, τ^2^ = 0.00, Q = 1.0 (*p* = 0.080)) /	3 0	15 (0.14 to 0.17, I^2^ = 0%, τ^2^ = 0.00, Q = 2.0 (*p* = 0.090)) /	3 0 3	69 (0.68 to 0.71, I^2^ = 0%, τ^2^ = 0.00, Q = 3.0 (*p* = 0.220)) /
Study year Before 2015 After 2015	5 7	48 (0.46 to 0.49, I^2^ = 0% τ^2^ = 0.00, Q = 4.0 (*p* = 0.320)) 78 (0.76 to 0.79, I^2^ = 0% τ^2^ = 0.00, Q = 6.0 (*p* = 0.530))	2 3	54 (0.50 to 0.58, I^2^ = 0%, τ^2^ = 0.00, Q = 1.0 (*p* = 0.070)) 57 (0.56 to 0.58, I^2^ = 0%, τ^2^ = 0.00, Q = 2.0 (*p* = 0.100))	2 3	15 (0.13 to 0.17, I^2^ = 0%, τ^2^ = 0.00, Q = 1.0 (*p* = 0.060)) 68 (0.65 to 0.71, I^2^ = 0% τ^2^ = 0.00, Q = 2.0 (*p* = 0.110))	3 2	74 (0.73 to 0.76, I^2^ = 0% τ^2^ = 0.00, Q = 2.0 (*p* = 0.130)) 36 (0.33 to 0.38, I^2^ = 0%, τ^2^ = 0.00, Q = 1.0 (*p* = 0.060))

NE: not estimable; /: no outcome.

## Data Availability

The original contributions presented in the study are included in the article/Supplementary Materials, further inquiries can be directed to the corresponding author.

## Supplementary Materials

The following supporting information can be downloaded at: https://www.mdpi.com/article/10.3390/healthcare12212131/s1, Table S1: Risk Of Bias In Non-randomised Studies-of Interventions (ROBINS-I); Table S2: Quality scores of the studies included in the meta-analysis, assessed by the Newcastle-Ottawa scale; Figure S1: Funnel plot for awareness of UCB by pregnant women; Figure S2: Subgroup analysis of primary outcome by region; Supplementary File S1. Detailed search strategy; Supplementary File S2. Questions and/or text from included studies with reported outcomes of interest; Supplementary File S3. Changes to the Systematic Review Protocol (PROSPERO CRD42023484499).
