# Supplementary material for: Knowledge, Attitudes, and Practices of Pregnant Women and Hospital Staff Regarding Umbilical Cord Blood Banking: Systematic Review and Meta-Analysis
Source: Healthcare (Basel). 2024 Oct 25;12(21):2131. doi: 10.3390/healthcare12212131 (PMC11544813; doi:10.3390/healthcare12212131)
Supplement: Supplementary file 1 [file healthcare-12-02131-s001.zip › 3 - Table S1- ROBINS-I.pdf]

Table S1. Risk Of Bias In Non-randomised Studies-of Interventions (ROBINS-I)

**Study**	Bias due to confounding	Bias in selection of participants into the study	Bias in classification of interventions	Bias due to deviations from intended interventions	Bias due to missing data	Bias in measurement of outcomes	Bias in the selection of reported results	Overall
Dinc [23]	?	+	+	?	+	?	+	+
Matijevic [25]	?	+	+	?	?	?	+	+
Fernandez [30]	?	+	+	+	?	?	?	+
Armstrong [28]	+	?	+	?	?	+	?	+
Bhandari [26]	?	?	+	+	?	?	?	?
Screnci [31]	?	+	+	+	?	+	+	+
Debiazi Zomer [27]	?	+	+	+	?	?	?	+
Grano [40]	+	+	+	+	x	?	?	+
Szubert [41]	?	+	+	+	?	x	x	?
Abdulrazeq [32]	?	?	+	?	?	?	?	+
Jordens [33]	?	+	+	+	+	+	+	+
Katz [34]	+	+	+	+	?	+	?	+
Saleh [35]	?	+	+	+	x	?	?	?
Thornley [36]	x	?	+	+	?	?	x	+
Palten[37]	?	+	+	?	?	x	?	?
Pandey [38]	?	?	+	+	+	+	+	+
Mayfield [39]	+	?	+	+	?	+	+	+
Tuteja [24]	+	+	+	?	x	+	?	+

Walker [29]	?	+	+	+	+	?	?	+

+ Low risk of bias

? Moderate risk of bias

x Serious risk of bias

! Critical risk of bias
