# Supplementary material for: Knowledge, Attitudes, and Practices of Pregnant Women and Hospital Staff Regarding Umbilical Cord Blood Banking: Systematic Review and Meta-Analysis
Source: Healthcare (Basel). 2024 Oct 25;12(21):2131. doi: 10.3390/healthcare12212131 (PMC11544813; doi:10.3390/healthcare12212131)
Supplement: Supplementary file 1 [file healthcare-12-02131-s001.zip › 4 - Table S2- NOS.pdf]

Table S2. Quality scores of the studies included in the meta-analysis, assessed by the Newcastle-Ottawa scale.

		**Selection**				**Comparability [a]**	**Outcome**			**Overall quality**
**Author**	**Year**	**Representativeness of the exposed cohort**	**Selection of the non-exposed cohort**	**Ascertainment of exposure**	**Demonstration that the outcome of interest was not present at the start of the study**	**Comparability of cohorts on the basis of the design or analysis**	**Assessment of the outcome**	**Was follow-up enough for outcomes to occur**	**Adequacy of follow-up of cohorts**	
Dinc	2009	*	*	*	*	**	*	*	*	**9**
Matijevic	2016	*	*	*	*	0	*	*	*	**7**
Fernandez	2003	*	*	*	*	0	*	0	*	**6**
Armstrong	2017	*	*	*	*	0	0	*	*	**6**
Bhandari	2016	*	*	0	*	**	*	*	*	**8**
Screnci	2012	*	*	*	*	**	*	*	*	**9**
Debiazi Zomer	2021	*	*	*	*		0	*	*	**6**
Grano	202	*	*	*	*	0	*	*	*	**7**

	0									
Szubert	2020	*	*	*	*	0	*	*	*	**7**
Abdulrazeq	2019	*	*	0	*	**	*	*	*	**8**
Jordens	2014	*	*	*	*	0	*	*	*	**7**
Katz	2011	*	*	*	*	0	0	*	*	**6**
Saleh	2019	*	*	*	*	0	*	*	*	**7**
Thornley	2009	*	*	0	*	**	*	*	*	**8**
Palten	2010	*	*	*	*	**	*	*	*	**9**
Pandey	2016	*	*	0	*	**	0	*	*	**7**
Mayfield	2023	0	*	*	*	0	*	*	*	**6**
Tuteja	2015	*	*	*	*	0	0	*	*	**6**
Walker	2012	*	*	0	*	0	*	*	*	**6**

Newcastle-Ottawa scale for assessment of quality of included studies - cohort studies (each asterisk represents if individual criterion within the subsection was fulfilled). [a] Comparability of cohorts: for the most important factors: age, education level, and occupation
