# Supplementary material for: Knowledge, Attitudes, and Practices of Pregnant Women and Hospital Staff Regarding Umbilical Cord Blood Banking: Systematic Review and Meta-Analysis
Source: Healthcare (Basel). 2024 Oct 25;12(21):2131. doi: 10.3390/healthcare12212131 (PMC11544813; doi:10.3390/healthcare12212131)
Supplement: Supplementary file 1 [file healthcare-12-02131-s001.zip › 5 - Supplementary File S1.pdf]

Supplementary File S1. Detailed search strategy

Database search results (from inception to November 30th, 2023): 330

MEDLINE (accessed through Pubmed) (from inception to November 30th, 2023): 80

(("fetal blood"[MeSH Terms] OR ("fetal"[All Fields] AND "blood"[All Fields]) OR "fetal blood"[All Fields] OR ("umbilical"[All Fields] AND "cord"[All Fields] AND "blood"[All Fields]) OR "umbilical cord blood"[All Fields]) AND ("bank s"[All Fields] OR "banked"[All Fields] OR "banking"[All Fields] OR "banks"[All Fields]) AND ("knowledge"[MeSH Terms] OR "knowledge"[All Fields] OR "knowledge s"[All Fields] OR "knowledgeability"[All Fields] OR "knowledgeable"[All Fields] OR "knowledgeably"[All Fields] OR "knowledges"[All Fields] OR ("attitude"[MeSH Terms] OR "attitude"[All Fields] OR "attitudes"[All Fields] OR "attitude s"[All Fields]) OR ("inform"[All Fields] OR "informal"[All Fields] OR "informality"[All Fields] OR "informally"[All Fields] OR "informant"[All Fields] OR "informant s"[All Fields] OR "informants"[All Fields] OR "information"[All Fields] OR "information s"[All Fields] OR "informational"[All Fields] OR "informations"[All Fields] OR "informative"[All Fields] OR "informatively"[All Fields] OR "informativeness"[All Fields] OR "informativity"[All Fields] OR "informed"[All Fields] OR "informer"[All Fields] OR "informers"[All Fields] OR "informing"[All Fields] OR "informs"[All Fields]))) AND (classicalarticle[Filter] OR clinicalstudy[Filter] OR comparativestudy[Filter] OR evaluationstudy[Filter] OR observationalstudy[Filter] OR researchsupportamericanrecoveryandreinvestmentact[Filter] OR researchsupportnihextramural[Filter] OR researchsupportnihintramural[Filter] OR researchsupportnonusgovt[Filter] OR researchsupportusgovtnonphs[Filter] OR researchsupportusgovtphs[Filter] OR researchsupportusgovernment[Filter])

EMBASE (from inception to November 30th, 2023): 150

('umbilical cord blood banking' OR (('umbilical'/exp OR umbilical) AND cord AND ('blood'/exp OR blood) AND banking)) AND ('knowledge'/exp OR knowledge OR 'attitude'/exp OR attitude OR 'information'/exp OR information) AND 'article'/it

Scopus: 100

TITLE-ABS-KEY (umbilical AND cord AND blood AND banking AND (knowledge OR attitude OR information)) AND PUBYEAR > 1991 AND PUBYEAR < 2024 AND (LIMIT-TO (DOCTYPE , "ar"))

Scielo.br (from inception to November 30th, 2023): 0
umbilical cord blood banking and (knowledge or attitude or information)

PROSPERO (from inception to November 30th, 2023): 0
umbilical cord blood banking and (knowledge or attitude or information)

CINAHL (from inception to November 30th, 2023):0
umbilical cord blood banking and (knowledge or attitude or information)

PsycINFO (from inception to November 30th, 2023): 0
umbilical cord blood banking and (knowledge or attitude or information)

AMED (from inception to November 30th, 2023): 0
umbilical cord blood banking and (knowledge or attitude or information)

LILACS (from inception to November 30th, 2023): 0

umbilical cord blood banking and (knowledge or attitude or information)

Duplicate removal (Endnote “Find duplicate” features): 87

Excluded articles with reason for exclusion after full text assessment

Article	Reason for exclusion
1. Peberdy L, Young J, Massey DL, Kearney L. Parents' knowledge, awareness and attitudes of cord blood donation and banking options: an integrative review. BMC Pregnancy Childbirth. 2018 Oct 10;18(1):395. doi: 10.1186/s12884-018-2024-6.	Review
2. Pisula A, Sienicka A, Stachyra K, Kacperczyk-Bartnik J, Bartnik P, Dobrowolska-Redo A, Romejko-Wolniewicz E. Women's attitude towards umbilical cord blood banking in Poland. Cell Tissue Bank. 2021 Dec;22(4):587-596. doi: 10.1007/s10561-021-09914-y.	No population of interest
3. Karagiorgou LZ, Pantazopoulou MN, Mainas NC, Beloukas AI, Kriebardis AG. Knowledge about umbilical cord blood banking among Greek citizens. Blood Transfus. 2014 Jan;12 Suppl 1(Suppl 1):s353-60. doi: 10.2450/2013.0297-12. Epub 2013 Oct 3.	No population of interest
4. Petrini C. Ethical issues in umbilical cord blood banking: a comparative analysis of documents from national and international institutions. Transfusion. 2013 Apr;53(4):902-10. doi: 10.1111/j.1537-2995.2012.03824.x. Epub 2012 Jul 31.	Commentary
5. Armson BA, Allan DS, Casper RF. Umbilical Cord Blood: Counselling, Collection, and Banking. J Obstet Gynaecol Can. 2015 Sep;37(9):832-844. doi: 10.1016/S1701-2163(15)30157-2.	Review
6. Fox NS, Chervenak FA, McCullough LB. Ethical considerations in umbilical cord blood banking. Obstet Gynecol. 2008 Jan;111(1):178-82. doi: 10.1097/01.AOG.0000295935.29407.4b.	Commentary
7. Gupta V, Agarwal L, Ballal P, Pandey D. Cord Blood Banking: Antenatal Care	Out of topic

Provider's Roles and Responsibilities. Stem Cells Int. 2019 Mar 7;2019:3598404. doi: 10.1155/2019/3598404.	
8. Matsumoto MM, Dajani R, Khader Y, Matthews KR. Assessing women's knowledge and attitudes toward cord blood banking: policy and ethical implications for Jordan. Transfusion. 2016 Aug;56(8):2052-61. doi: 10.1111/trf.13650.	No population of interest
9. Grieco D, Lacetera N, Macis M, Di Martino D. Motivating Cord Blood Donation with Information and Behavioral Nudges. Sci Rep. 2018 Jan 10;8(1):252. doi: 10.1038/s41598-017-18679-y.	Out of topic
10. Parco S, Vascotto F, Visconti P. Public banking of umbilical cord blood or storage in a private bank: testing social and ethical policy in northeastern Italy. J Blood Med. 2013 Apr 10;4:23-9. doi: 10.2147/JBM.S41532.	Out of topic
11. Herlihy MM, Delpapa EH. Obstetricians and their role in cord blood banking: promoting a public model. Obstet Gynecol. 2013 Apr;121(4):851-855. doi: 10.1097/AOG.0b013e31828882aa.	Commentary
12. Marcon AR, Murdoch B, Caulfield T. Peddling promise? An analysis of private umbilical cord blood banking company websites in Canada. Cell Tissue Bank. 2021 Dec;22(4):609-622. doi: 10.1007/s10561-021-09919-7.	Out of topic
13. Broder SM, Ponsaran RS, Goldenberg AJ. US public cord blood banking practices: recruitment, donation, and the timing of consent. Transfusion. 2013 Mar;53(3):679-87. doi: 10.1111/j.1537-2995.2012.03785.x.	Out of topic
14. Manegold G, Meyer-Monard S, Tichelli A, Granado C, Hösli I, Troeger C. Controversies in hybrid banking: attitudes of Swiss public umbilical cord blood	No outcome of interest

donors toward private and public banking. Arch Gynecol Obstet. 2011 Jul;284(1):99-104. doi: 10.1007/s00404-010-1607-x.	
15. Perlow JH. Patients' knowledge of umbilical cord blood banking. J Reprod Med. 2006 Aug;51(8):642-8. PMID: 16967635.	Unretrievable Full-text
16. Peberdy L, Young J, Massey D, Kearney L. Maternity health professionals' perspectives of cord clamp timing, cord blood banking and cord blood donation: a qualitative study. BMC Pregnancy Childbirth. 2020 Jul 16;20(1):410.	Out of topic
17. Danzer E, Holzgreve W, Troeger C, Kostka U, Steimann S, Bitzer J, Gratwohl A, Tichelli A, Seelmann K, Surbek DV. Attitudes of Swiss mothers toward unrelated umbilical cord blood banking 6 months after donation. Transfusion. 2003 May;43(5):604-8. doi: 10.1046/j.1537-2995.2003.00375.x.	No population of interest
