# Supplementary material for: Knowledge, Attitudes, and Practices of Pregnant Women and Hospital Staff Regarding Umbilical Cord Blood Banking: Systematic Review and Meta-Analysis
Source: Healthcare (Basel). 2024 Oct 25;12(21):2131. doi: 10.3390/healthcare12212131 (PMC11544813; doi:10.3390/healthcare12212131)
Supplement: Supplementary file 1 [file healthcare-12-02131-s001.zip › 6 - Supplementary File S2.pdf]

Supplementary File S2. Questions and/or text from included studies with reported outcomes of interest

Awareness rate (pregnant women)

**Study**	**Text**
Dinç 2009, Turkey	*Do you have information about stem cells and cord*
Matijevic 2016, Croatia	*Are you familiar with the possibility of UCB banking?*
Fernandez 2003, Canada	*Rated knowledge good*
Screnci 2012, Italy	*General information about CB*
Debiazi Zomer 2021, Brazil	*ever heard about UCB banking,*
Grano 2020, Italy	*being aware of the possibility of donating or conserving UCB*
Szubert 2020, Poland	*I've heard of it and consider this procedure useful or useless*
Jordens 2014, Australia	*aware of cord blood banking.*
Katz 2011, Europe	*declare having a basic knowledge of cord blood banking*
Pandey 2016, India	*Banking of blood from the placental side 68 (26.5%) of the cord*
Mayfield 2023, USA	*Have you ever heard of cord blood before taking this survey*
Saleh, 2019, Lebanon	*Knowledge of UCB stem cells banking*

Awareness rate (hospital staff)

**Study**	**Text**
Matijevic 2016, Croatia	*Are you familiar with the possibility of UCB banking?*
Armstrong 2017, USA	*Do you discuss UCB donation during prenatal visits?*
Bhandari 2016, USA	*Do you discuss UCB donation during prenatal visits?*
Tuteja 2015, India	*Have you heard of UCB?*
Walker 2012, USA	*familiar with the ACOG position statement on UCB banking*

Positive attitude (pregnant women)

**Study**	**Text**
Dinç 2009, Turkey	*Do you think about storing your cord blood?*
Debiazi Zomer 2021, Brazil	*would consider donating their child's UCB to a public bank*
Szubert 2020, Poland	*I have heard of it and consider this procedure to be useful*
Palten 2010, Germany	*Page 652 (UCBB is rarely…)*
Pandey 2016, India	*Likelihood of using the stored If 2500 have stored one might need it 46 (18.1%) cord blood stem cells in future*

Plan to store UCB (pregnant women)

Study	Text
Matijevic 2016, Croatia	Are you planning to store UCB after delivery?
Screnci 2012, Italy	Intention to private storage
Debiazi Zomer 2021, Brazil	Only 13% of the expecting mothers had already decided whether to store or donate their child's UCB
Jordens 2014, Australia	Would consider donating or storing cord blood
Katz 2011, Europe	Would store in a public or private bank

Research purpose (pregnant women)

Study	Text
Dinç 2009, Turkey	I am concerned that my baby's cord blood would be used for different purposes.
Debiazi Zomer 2021, Brazil	would allow its use for medical research
Jordens 2014, Australia	would be agreeable for the blood to be used for medical research.
Saleh 2019, Lebanon	Would donate UCB stem cells for research purposes,
Pandey 2016, India	would like to bank cord blood for research
