# Supplementary material for: Knowledge, Attitudes, and Practices of Pregnant Women and Hospital Staff Regarding Umbilical Cord Blood Banking: Systematic Review and Meta-Analysis
Source: Healthcare (Basel). 2024 Oct 25;12(21):2131. doi: 10.3390/healthcare12212131 (PMC11544813; doi:10.3390/healthcare12212131)
Supplement: Supplementary file 1 [file healthcare-12-02131-s001.zip › 7 - Supplementary File S3.pdf]

Changes to the Systematic Review Protocol (PROSPERO CRD42023484499)

Title:
Knowledge, attitude and practice of pregnant women and hospital staff about umbilical cord blood banking: systematic review and meta-analysis.

Review question
Changed patients to “pregnant patients” and healthcare workers” to “hospital staff”

Searches
Cochrane CENTRAL was not searched due to absence of RCTs in the eligibility criteria.

Participants/population
Non-pregnant women were excluded to avoid heterogeneity

Measures of effect
Effect size estimates only were used due to observational nature of studies

Risk of bias (quality) assessment
ROBINS-I was used to Risk of bias assessment due to observational nature of studies

Strategy for data synthesis

Due to observational nature of studies, a random-effects restricted maximum-likelihood model with Freeman-Tukey Double arcsine transformation meta-analysis was carried out to calculate pooled effect size estimates. MOOSE guidelines were followed. STATA 14.1 was used for statistical analysis.
