# Supplementary figures and images for: Knowledge, Attitudes, and Practices of Pregnant Women and Hospital Staff Regarding Umbilical Cord Blood Banking: Systematic Review and Meta-Analysis
Source: Healthcare (Basel). 2024 Oct 25;12(21):2131. doi: 10.3390/healthcare12212131 (PMC11544813; doi:10.3390/healthcare12212131)

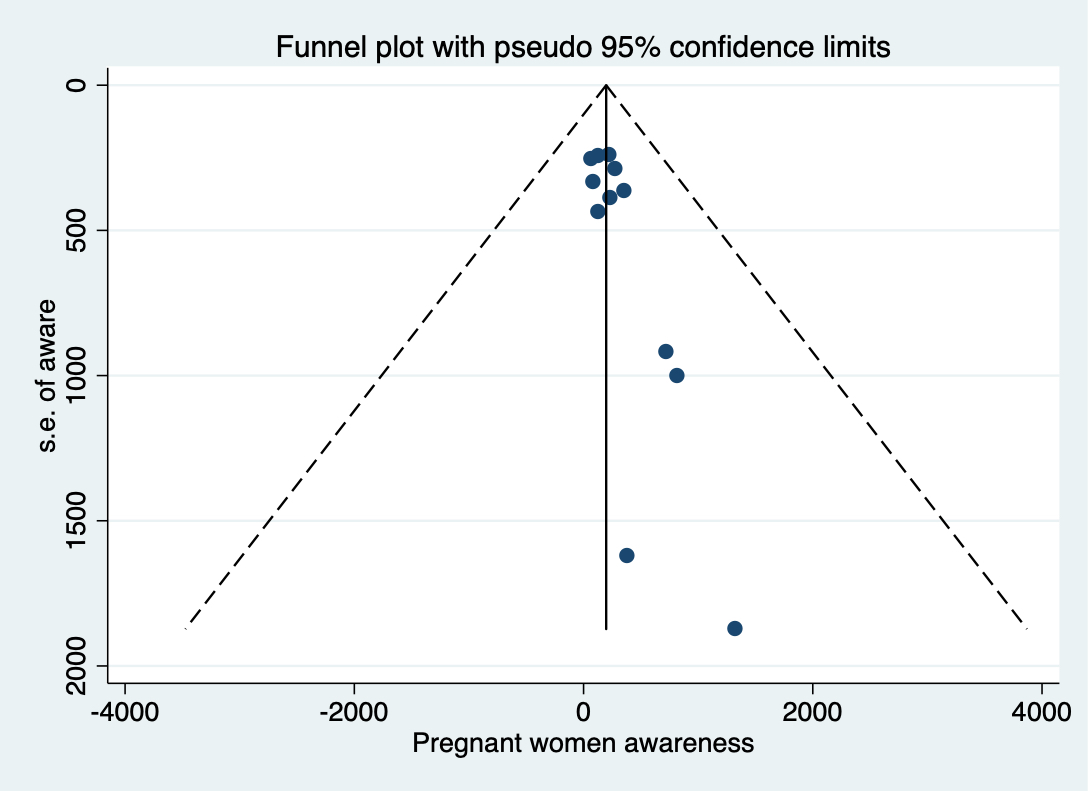

Supplement: Supplementary file 1 [file healthcare-12-02131-s001.zip › 1 - Figure S1 - funnel.jpg]

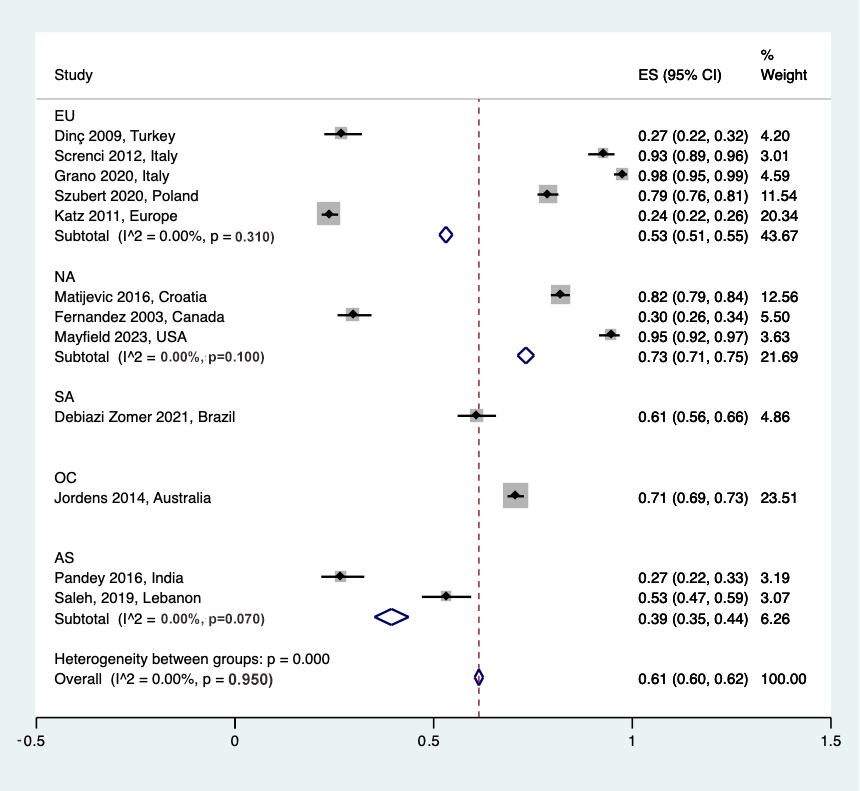

Supplement: Supplementary file 1 [file healthcare-12-02131-s001.zip › 2 - Figure S2- region.tif]
